# Surface Ion-Imprinted Polypropylene Fibers for Selective and Rapid Adsorption of Borate Ions: Preparation, Characterization, and Performance Study

**DOI:** 10.3390/polym17101368

**Published:** 2025-05-16

**Authors:** Hui Jiang, Xinchi Zong, Zhengwei Luo, Wenhua Geng, Jianliang Zhu

**Affiliations:** 1 College of Biotechnology and Pharmaceutical Engineering, Nanjing Tech University, Nanjing 211816, China; huijiang@njtech.edu.cn (H.J.); 202261118073@njtech.edu.cn (X.Z.); jlzhu@njtech.edu.cn (J.Z.); 2 School of Environmental Science and Engineering, Nanjing Tech University, Nanjing 211816, China; luozw2015@njtech.edu.cn

**Keywords:** ion imprinting technology, plasma, graft polymerization, boron, selectivity of adsorption

## Abstract

This study presents a novel ion-imprinted fiber material, I-(PP-g-GMA-NMDG), designed for the rapid and selective adsorption of borate ions. Leveraging low-temperature plasma graft polymerization, polypropylene (PP) melt-blown fibers were functionalized with glycidyl methacrylate (GMA) and *N*-methyl-*D*-glucamine (NMDG) to introduce tailored recognition sites. Systematic optimization of plasma parameters (100 W discharge power, O_2_ atmosphere) and liquid-phase grafting conditions (28.5% GMA, 85 °C, 2.5 h) achieved a grafting rate of 203.26%. The imprinted fibers exhibited exceptional adsorption performance, with a maximum capacity of 35.85 mg/g at pH 9, reaching 90% saturation within 60 min. Adsorption kinetics adhered to a pseudo-second-order model, while the Freundlich isotherm indicated multilayer adsorption. Competitive ion experiments demonstrated high selectivity for B(OH)_4_^−^ over anions (SO_4_^2−^ and Cl^−^) and cations (Na^+^, K^+^, Ca^2+^, and Mg^2+^), which was attributed to the precise spatial and charge complementarity of the imprinted cavities. Characterization via FT-IR, XRD, and SEM confirmed successful synthesis and structural stability. The material retained 78.1% adsorption efficiency after five regeneration cycles, showcasing its practicality for boron recovery from wastewater. This work advances boron-selective adsorption technology by combining plasma modification with ion imprinting, offering a sustainable solution for industrial and environmental applications.

## 1. Introduction

Boron (B) is a strategic resource with irreplaceable roles in high-tech industries (e.g., semiconductors and nuclear energy) and glass, ceramics, metallurgy, agriculture, and the medicine industry [1,2,3,4,5]. While China possesses abundant salt boron reserves, inefficient extraction technologies and heavy reliance on imports hinder its utilization [6]. Traditional extraction methods (e.g., acidification crystallization and solvent extraction) suffer from low efficiency, high reagent consumption, and secondary pollution [7]. Particularly, the resulting wastewater often exceeds WHO safety thresholds (0.5 mg/L for drinking water; 1.0 mg/L for irrigation), posing severe environmental risks. These challenges necessitate green technologies for boron recovery and wastewater purification [8].

Recent advances in adsorption and membrane separation offer promising alternatives to conventional approaches. However, most adsorbents face practical limitations: granular/powdered forms require energy-intensive separation (e.g., centrifugation and magnetic recovery), while polymeric fibers with a large surface area often lack tailored functionality. Polypropylene (PP) melt-blown fibers, with their mechanical robustness, microporous structure, and ease of scale-up, represent an ideal substrate for functionalization. However, their non-polar surface inherently lacks boron-binding groups (e.g., vicinal diols), limiting direct application. To address this, grafting strategies (e.g., radiation and chemical modification) have been explored but compromise fiber integrity or involve toxic reagents [9,10,11,12].

Herein, we propose a plasma-enabled ion imprinting strategy to engineer polypropylene (PP) fibers with borate-selective recognition sites, synergistically integrating low-temperature plasma polymerization for bulk property preservation and molecular imprinting for specificity enhancement. This bifunctional adsorbent achieves targeted affinity through the precise grafting of cis-diol motifs matching the borate coordination geometry, ensures structural stability by avoiding fiber crystallinity damage associated with conventional radiation methods [13,14,15], and offers operational practicality via the macroscopic fiber morphology enabling rapid solid–liquid separation without energy input. Advancing boron recovery technology, this work provides scientific innovation through mechanistic insights into plasma-induced surface reconstruction and imprinting efficiency, and achieves a technical breakthrough with a reagent-less protocol outperforming multi-step chemical grafting [15,16,17,18,19,20]. It holds promising prospects in key scenarios such as the efficient recovery of strategic mineral resources, the utilization of industrial wastewater, and the advanced treatment of trace pollutants.

## 2. Experimental

### 2.1. Experimental Materials

PP melt-blown fibers were obtained from School of Textile Science and Engineering, Jiangnan University. Anhydrous ethanol and anhydrous methanol were obtained from Yasheng Chemical Co., Ltd. (AR, Wuxi, China). Acetone and isopropanol were obtained from China National Pharmaceutical Group Chemical Reagent Co., Ltd. (AR, Shanghai, China). GMA, *N*,*N*-dimethylformamide and glutaraldehyde were obtained from Aladdin Biochemical Technology Co., Ltd. (AR, Shanghai, China). 1,4-Dioxane, epichlorohydrin and boric acid were obtained from Lingfeng Chemical Reagent Co., Ltd. (AR, Shanghai, China). N-Methyl-D-glucosamine was obtained from Yuanye Biotechnology Co., Ltd. (AR, Shanghai, China). Hydrochloric acid was obtained from Yonghua Chemical Co., Ltd. (AR, Shanghai, China).

### 2.2. Experimental Instruments

The plasma generator used in this study was VP-R3 vacuum plasma processor manufactured by Shanzhun Technology Co., Ltd. (Shenzhen, China).

### 2.3. Experimental Methods

#### 2.3.1. Material Pretreatment

Cut PP fibers into dimensions of 5.0 cm × 5.0 cm, with an approximate mass of 0.1 g. Subsequently, immerse the fibers sequentially in ethanol and deionized water, followed by cleaning them using ultrasonic waves at room temperature for a duration of 1 h. Finally, dry the fibers for future applications.

#### 2.3.2. Plasma Modification

Weigh 0.1 g of dried PP fiber and place it within a plasma reaction chamber. Select the appropriate discharge power (40 W, 100 W, or 160 W), initiate the plasma apparatus, evacuate the chamber, and introduce the reaction gas (Ar, N_2_, O_2_, or air). Adjust the gas flow rate accordingly (100–300 mL/min) and maintain the discharge for a predetermined duration (0–30 min) to facilitate the reaction. Subsequently, evaluate the effects of plasma modification on the PP fiber samples.

#### 2.3.3. Liquid-Phase Grafting Treatment

Following plasma treatment, remove the PP fibers and immerse them in a methanol solution containing GMA (5–30%). Add ferrous ammonium sulfate and conduct the liquid-phase grafting reaction at a specified temperature (60–95 °C) for several hours (0.5–3 h). Once the reaction is complete, clean the fiber surface to remove any unreacted GMA monomers and homopolymers using acetone. Subsequently, wash the fibers with deionized water and dry them in a vacuum oven until a constant weight is achieved, resulting in the modified PP-g-GMA fiber sample.

#### 2.3.4. The Ring-Opening Amination Reaction

The ring-opening amination reaction involves placing 0.05 g of PP-g-GMA fibers and a specific weight of NMDG (0.05–3 g) in a designated volume of solvent (isopropanol, *N*,*N*-dimethylformamide, or 1,4-dioxane). The mixture is then shaken in a water bath at an appropriate temperature (80–85 °C) for a specified duration (0.5–12 h). Upon the completion of the experiment, the material is removed, washed several times, and vacuum-dried to a constant weight, thereby yielding PP-g-GMA-NMDG chelating fibers.

#### 2.3.5. Preparation of the Borate-Imprinted Fiber I-(PP-g-GMA-NMDG)

**Figure 1:**
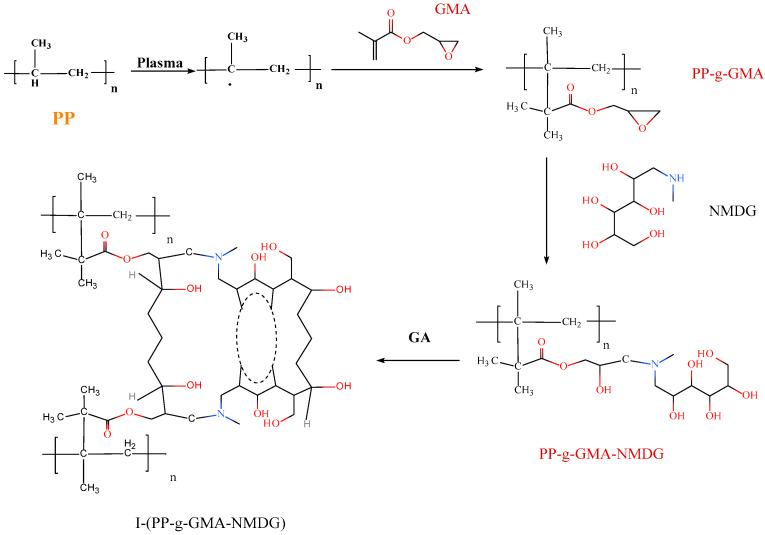
Flow chart of the preparation of I-(PP-g-GMA-NMDG) fibers.

A quantity of 0.03 g of PP-g-GMA-NMDG chelating fibers was utilized and subsequently loaded with borate ions to achieve adsorption equilibrium. Following this procedure, the fiber was removed and placed into a stoppered glass sample bottle. Subsequently, a specific volume (5–30 mL) of various crosslinking agents (epichlorohydrin, *N*,*N*-dimethylformamide, and glutaraldehyde) was added to the mixture. The crosslinking process was carried out in a water bath shaker at an appropriate temperature (50–70 °C) with a rotational speed of 140 r/min for a specified duration (1–5 h). After the elution process, the material was dried in preparation for subsequent adsorption experiments. The above preparation process is shown in Figure 1.

#### 2.3.6. Adsorption Experiment

Prepare a boric acid aqueous solution with a concentration of 100 mg/L to simulate a water sample. Weigh 0.01 g of the I-(PP-g-GMA-NMDG) fiber material and place it in a stoppered glass sample bottle. Subsequently, adjust the pH of the solution to the optimal conditions; then, add 10 mL of the boric acid aqueous solution and shake the bottle in a constant temperature water bath shaker set at 25 °C at a speed of 200 r/min for 2 h. Measure the boron concentration in the aqueous solution by performing membrane filtration on the solution before and after adsorption.

#### 2.3.7. Calculation of the Grafting Rate and Adsorption Amount

The grafting rate is calculated using the following formula:

(1) $$GP=\frac{\left(W_{1}-W_{0}\right)}{W_{0}}\times 100\%$$

where *W*_0_ represents the dry weight of the fibers before grafting, g; *W*_1_ denotes the dry weight of the fibers after grafting, g.

The aqueous solution before and after adsorption was filtered through a membrane, and the boron concentration in the water was measured by ICP-OES (iCAP 6300, Thermo Fisher, Waltham, MA, USA). Then, the adsorption capacity was calculated using the following formula.

(2) $$q_{t}=\frac{\left(C_{0}-C_{t}\right)V}{m}$$

where *q*_t_ represents the adsorption capacity of the I-(PP-g-GMA-NMDG) fibers for boron at time *t*, mg/g; *C*_0_ denotes the initial concentration of boron, mg/L; *C*_t_ indicates the residual boron concentration in the solution at time *t*, mg/L; *V* is the volume of the adsorption solution, L; and *m* is the mass of I-(PP-g-GMA-NMDG) fibers, g.

## 3. Results and Discussion

### 3.1. Optimization of the Low-Temperature Plasma Modification Conditions

This study employed low-temperature plasma grafting polymerization to modify PP fibers, which was conducted in two stages. Consequently, the conditions for the low-temperature plasma modification and liquid-phase grafting are discussed separately. To facilitate the exploration of low-temperature plasma modification conditions, the parameters for liquid-phase grafting were initially fixed: a reaction atmosphere of nitrogen (N_2_) gas, a reaction temperature of 70 °C, a reaction time of 2 h, and a reaction system consisting of 10 mL of GMA, 90 mL of anhydrous methanol, and 0.01 g of ferrous ammonium sulfate. The relevant factors investigated included the plasma discharge power, reaction atmosphere, gas flow rate, and discharge time.

#### 3.1.1. The Influence of Different Discharge Powers on GMA Grafting

**Figure 2:**
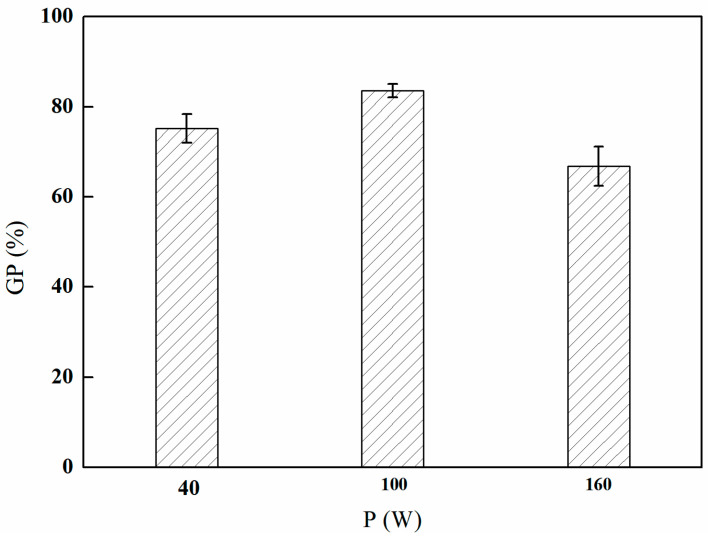
Effect of the plasma discharge power on GMA grafting (reaction atmosphere of Ar, discharge time of 5 min, and gas flow rate of 300 mL/min).

As depicted in Figure 2, the grafting rate of GMA exhibits an initial increase with discharge power, reaching a peak at 100 W, followed by a subsequent decline. This phenomenon can be explained by the fact that an increase in power within a specific range enhances both the energy and the concentration of active species (such as free radicals and ions) present in the plasma, thereby facilitating grafting reactions on the surface of the material and resulting in an increased grafting rate. However, as the plasma power continues to escalate, excessive energy may adversely affect the grafting reaction by inducing molecular chain scission or excessive etching of the surface materials, ultimately leading to a decrease in the grafting rate. Therefore, it was concluded that 100 W represents the optimal discharge power for the purposes of this study.

#### 3.1.2. The Influence of Different Atmospheres on GMA Grafting

**Figure 3:**
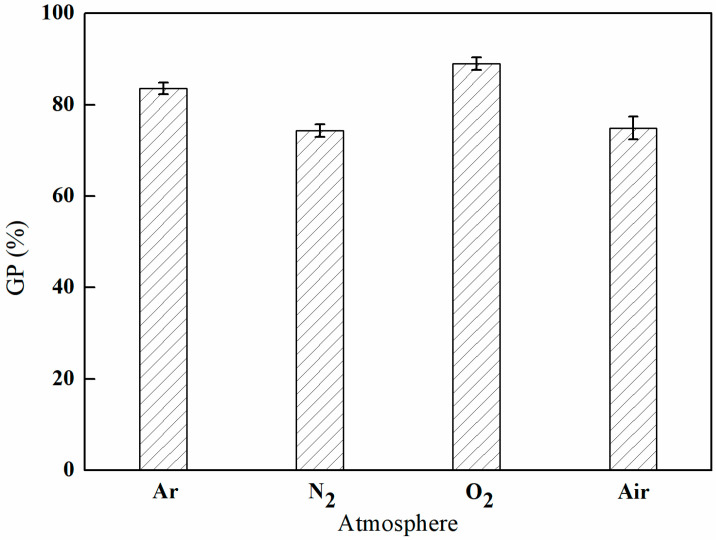
Effect of the plasma atmosphere on GMA grafting (power of 100 W, discharge time of 5 min, and gas flow rate of 300 mL/min).

As depicted in Figure 3, the grafting rate of GMA reached a peak of 89.02% in an oxygen (O_2_) atmosphere, while it was recorded at a low of 74.36% in a nitrogen (N_2_) atmosphere. This variation can be ascribed to the elevated reactivity of oxygen, which, under plasma excitation, produces highly reactive species, including oxygen radicals, superoxide radicals, and ozone. These reactive species play a crucial role in cleaving chemical bonds on the surfaces of materials, thereby initiating the grafting reactions. Furthermore, the introduction of oxygen-containing functional groups, such as hydroxyl and carboxyl groups, onto the surface of the material enhances both its reactivity and energy, thereby facilitating the grafting process.

#### 3.1.3. The Effect of Different Discharge Times on GMA Grafting

**Figure 4:**
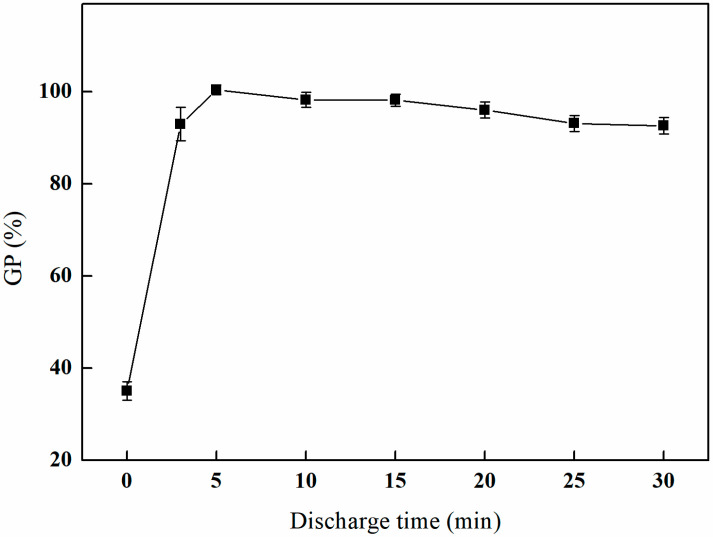
Effect of the plasma discharge time on GMA grafting (power of 100 W, reaction atmosphere of O_2_, and gas flow rate of 200 mL/min).

As depicted in Figure 4, the initial five minutes of the experiment reveal a rapid increase in the grafting rate, suggesting a heightened activity of the surface grafting reaction during this period. The grafting rate reaches a maximum of 100.41% at the five-minute interval. Subsequently, the grafting rate stabilizes at a consistently high level, exhibiting minor fluctuations within this range, while overall maintaining a value exceeding 90%.

### 3.2. Optimization of the Liquid-Phase Grafting Conditions

In accordance with the plasma modification optimization conditions outlined above (discharge power of 100 W, reaction atmosphere of O_2_, gas flow rate of 200 mL/min, and discharge time of 5 min), the parameters for liquid-phase grafting were optimized.

#### 3.2.1. The Influence of Different Solvent Environments on GMA Grafting

**Figure 5:**
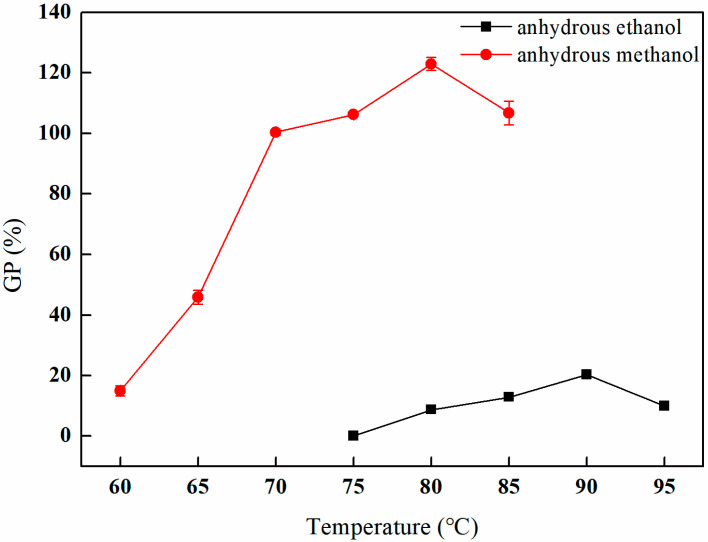
Effect of the solvent environment on GMA grafting (reaction atmosphere of N_2_, reaction duration of 2 h, and 10% GMA).

As depicted in Figure 5, within the anhydrous methanol solvent environment, the grafting rate exhibits a rapid increase as the temperature increases from 60 °C to 70 °C. The grafting rate continues to rise from 70 °C to 80 °C, albeit at a diminished rate, ultimately reaching a peak of 122.98% at 80 °C. However, further increases in temperature beyond this point result in a decline in the grafting rate. In contrast, when anhydrous ethanol is employed as the solvent, the grafting rate exhibits a gradual increase from 75 °C to 90 °C, achieving a maximum of 20.33% at 90 °C, after which a decline is also observed with continued temperature elevation. Notably, the grafting rate in the presence of anhydrous methanol remains comparatively high throughout the entire temperature range. This phenomenon can be attributed to the higher polarity of methanol relative to ethanol, which enhances the grafting reaction of GMA [21]. Furthermore, the comparatively lower boiling point of methanol relative to ethanol is a critical factor. Although elevated temperatures typically enhance molecular motion and promote chemical reactions, excessively high temperatures may also precipitate an increase in side reactions and solvent evaporation, ultimately impacting the grafting rate [22].

#### 3.2.2. The Effect of Different GMA Concentrations on GMA Grafting

**Figure 6:**
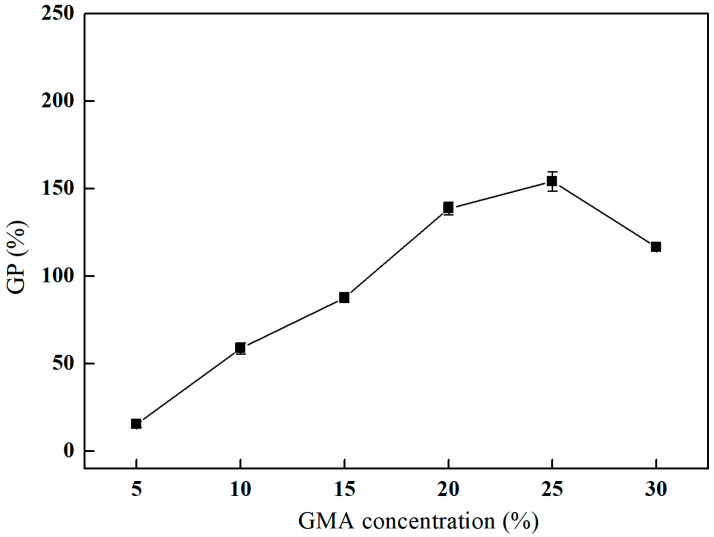
Effect of the GMA concentration on GMA grafting (reaction atmosphere of N_2_, reaction duration of 2 h, and reaction temperature of 80 °C).

The effect of varying concentrations of GMA on the grafting process was investigated. As depicted in Figure 6, the grafting rate exhibits a progressive increase with elevated GMA concentrations. The grafting rate reaches its maximum of 154.11% at a GMA concentration of 25%. However, with further increases in the GMA concentration, a decline in the grafting rate is observed [23], and partial dissolution of PP fibers is observed when the concentration of GMA reaches 35% or above. This phenomenon can be attributed to the increased availability of GMA molecules that can engage in the grafting reaction, which enhances the overall grafting yield. Nevertheless, as the concentration of GMA rises, the probability of competitive or side reactions occurring during the grafting process may also increase, resulting in a reduction in the grafting rate [24,25].

#### 3.2.3. The Effect of Different Grafting Times on GMA Grafting

**Figure 7:**
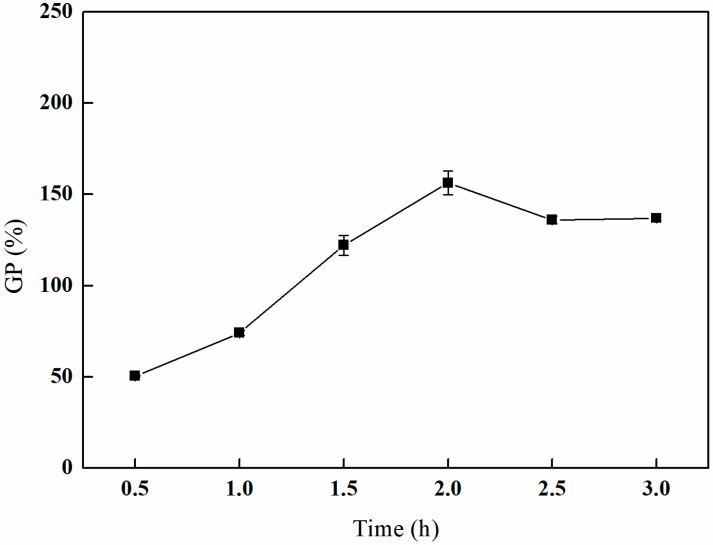
Effect of the grafting time on GMA grafting (reaction atmosphere of N_2_, 25% GMA, and reaction temperature of 80 °C).

As depicted in Figure 7, the grafting rate initially increases with an extended grafting time, subsequently decreases, and ultimately stabilizes. The peak grafting rate of 156.30% is attained after 2 h of grafting. This behavior can be attributed to the high concentration of reactants present in the early stages of the reaction, which facilitates a rapid reaction rate and a corresponding swift increase in the grafting rate. However, as the reaction time is extended, the concentration of reactants diminishes, leading to a reduction in the reaction rate and the potential occurrence of side reactions. Consequently, the reaction conditions become less favorable for the grafting process, resulting in a decline in the grafting rate [26,27].

### 3.3. Response Surface Optimization Experiment

A response surface experiment was developed utilizing the Box–Behnken design, which was guided by a single-factor experiment on liquid-phase grafting. In this design, the independent variables included the concentration of GMA, grafting temperature, and grafting time, while the grafting rate served as the response variable, as illustrated in Table 1. The experimental results are presented in Table 2.

Table 3 presents the results of the variance analysis for the response surface regression model. Statistical tests revealed that the model’s F-value was 7.16 (*p* = 0.0083 < 0.05), indicating the statistical significance of the model. The *p*-value for the mismatch term was 0.1074 (>0.05), suggesting a good fit between the model and the actual data. The corrected coefficient of determination was calculated to be 0.7760, signifying that the model can explain 77.60% of the variation in the response values, thereby exhibiting a strong predictive capability. The coefficient of variation (C.V.) was determined to be 10.50%, which is below the threshold requirement of 13%, thereby confirming the reliability of the experimental data and the stability of the model. The significance analysis of various factors indicated that the influences of the GMA concentration (A) and time (C) on the response value reached a statistically significant level (*p* < 0.05). Based on the sorting of F-values, the degree of influence of each factor on the GMA grafting rate is ranked as follows: time (C) > GMA concentration (A) > temperature (B) > interaction between temperature and time (BC) > interaction between GMA concentration and time (AC) > interaction between GMA concentration and temperature (AB).

**Figure 8:**
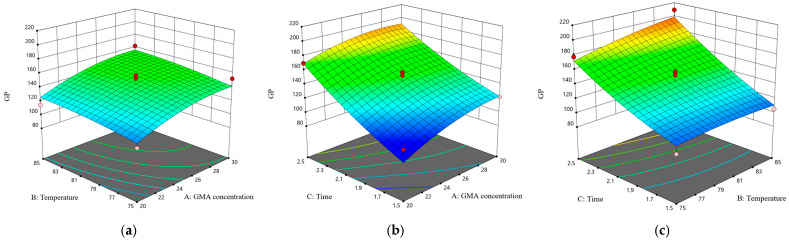
Response surface plots of the interactions between two factors on the grafting effect of GMA. (**a**) GMA concentration and temperature; (**b**) GMA concentration and time; and (**c**) temperature and time.

Furthermore, Figure 8 demonstrates the effects of the interactions between pairs of independent variables on the response variable. The data reveal that the interaction between temperature and time is the most pronounced, followed by the interaction between the GMA concentration and time, and, subsequently, the interaction between the GMA concentration and temperature. Through the optimization of the experimental conditions, the ideal parameters for the liquid-phase grafting of GMA were determined to be a GMA concentration of 28.5%, a grafting temperature of 85 °C, and a grafting duration of 2.5 h. Under these specified conditions, the model predicted a GMA grafting rate of 202.81%. In accordance with the aforementioned optimal process parameters for liquid-phase grafting, five experimental trials were conducted, resulting in an actual GMA grafting rate of 203.26 ± 2.12%, which closely corresponds with the predicted value generated by the model.

Multiple regression analyses were performed on the collected data to develop a polynomial model that describes the relationship between the independent variables and the response values.

*G* = −613.85125 + 20.36025 *X* + 12.72905 *Y* − 174.368 *Z* + 0.0248 *XY* − 1.571 *XZ* + 2.517 *YZ* − 0.32548 *X*^2^ − 0.10608 *Y*^2^ + 22.102 *Z*^2^ (3)

where *G* represents the grafting rate of GMA, %; *X* denotes the concentration of GMA, %; *Y* indicates the temperature, °C; and *Z* signifies the time, h.

### 3.4. Optimization of Open-Loop Amination Conditions

#### 3.4.1. The Influence of Different Solvent Environments on Amination Efficiency

**Figure 9:**
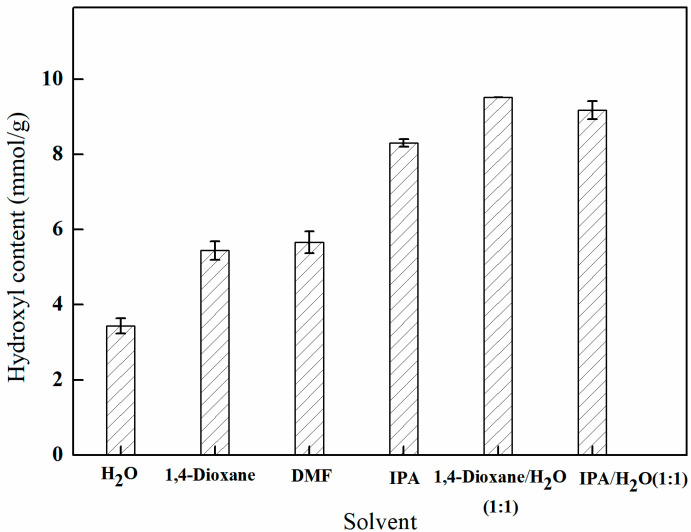
Effect of the solvent environment on amination (reaction duration of 8 h, temperature of 70 °C, and 1 g of NMDG).

As depicted in Figure 9, the efficacy of amination varies among the solvents, with the following ranking: isopropanol > *N*,*N*-dimethylformamide > 1,4-dioxane > water. Additionally, the utilization of a mixed solvent comprising both an organic solvent and water in specific proportions has a beneficial effect on amination. This observation suggests that the mixed solvent not only enhances the swelling of the PP-g-GMA fibers to a certain degree and facilitates mass transfer but also promotes the ring-opening reaction by functioning as a proton carrier. Consequently, different ratios of mixed solvents were selected for further analysis.

**Figure 10:**
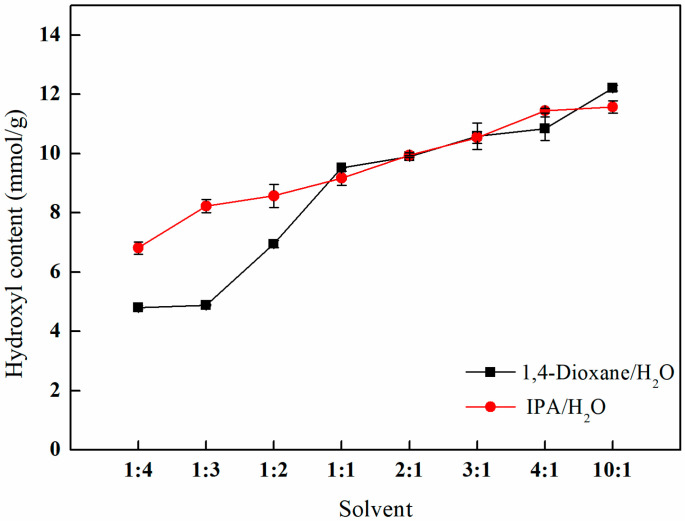
Effects of different proportions of mixed solvents on amination.

Figure 10 demonstrates the influence of varying ratios of 1,4-dioxane/water and isopropanol/water mixed solvents on the efficiency of amination. The results indicate a progressive increase in the hydroxyl content with the rising volume ratio of the mixed solvent. Notably, when the volume ratio of 1,4-dioxane/water to isopropanol/water reaches 10:1, the hydroxyl content attains its maximum values of 12.21 and 11.57 mmol/g, respectively. At this specific ratio, the amination effect is optimized. Additionally, considering the lower toxicity of isopropanol in comparison to 1,4-dioxane, a mixed solvent consisting of isopropanol and water at a volume ratio of 10:1 is selected as the solvent for the ring-opening reaction.

#### 3.4.2. The Effect of Different Open-Loop Times on Amination Efficiency

**Figure 11:**
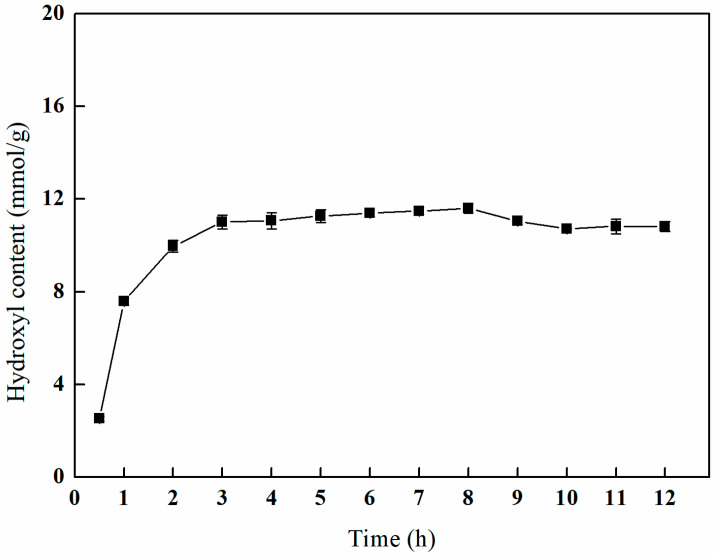
Effect of the reaction time on amination (V_IPA_/V_H2O_ = 10:1, temperature of 70 °C, and 1 g of NMDG).

As depicted in Figure 11, the hydroxyl content exhibits a gradual increase during the initial phase, followed by a more gradual change with a prolonged ring-opening time. After a reaction duration of 8 h, the hydroxyl content reaches a peak of 11.59 mmol/g. Following this peak, there is a slight decline in the hydroxyl content, which ultimately stabilizes. The data indicate that at a reaction time of 3 h, the hydroxyl content is measured at 11.01 mmol/g, a value that does not significantly differ from the observed maximum. Consequently, in light of cost efficiency considerations, a ring-opening duration of 3 h is deemed optimal.

#### 3.4.3. The Effect of Different Open-Loop Temperatures on Amination Efficiency

**Figure 12:**
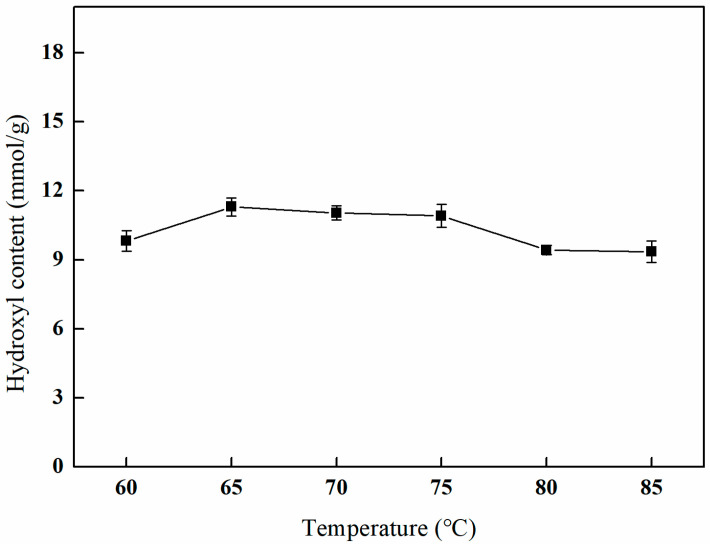
Effect of the reaction temperature on amination (V_IPA_/V_H2O_ = 10:1, reaction duration of 3 h, and 1 g of NMDG).

As depicted in Figure 12, the hydroxyl content initially increases with elevated reaction temperatures, subsequently decreases, and ultimately reaches a plateau. The peak hydroxyl content is observed at a reaction temperature of 65 °C. This behavior can be attributed to the fact that higher temperatures enhance the rate of the chemical reaction, thereby increasing hydroxyl content. However, excessively elevated temperatures may result in increased solvent evaporation and modifications to the fiber material, which can ultimately compromise the effectiveness of the amination process.

#### 3.4.4. The Influence of Different NMDG Amounts on the Amination Effect

**Figure 13:**
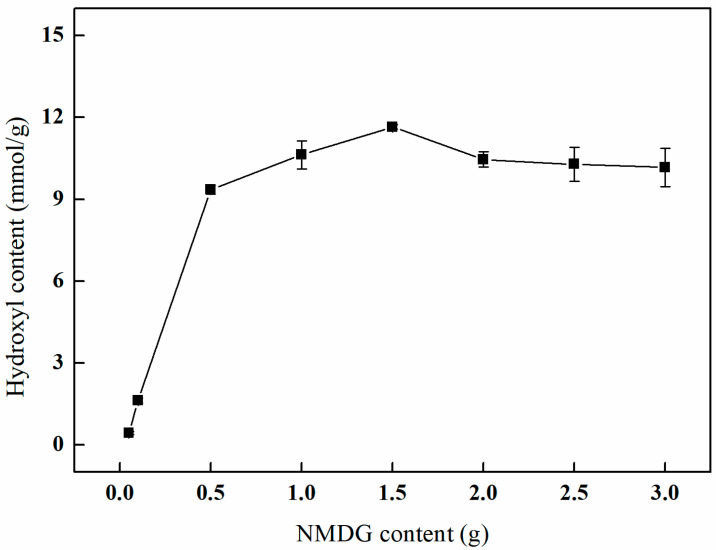
Effect of the NMDG content on amination (V_IPA_/V_H2O_ = 10:1, temperature of 65 °C, and reaction duration of 3 h).

As depicted in Figure 13, an increase in the concentration of NMDG is associated with a gradual enhancement in the hydroxyl content, which ultimately reaches a plateau. Specifically, when the concentration of NMDG attains 1.5 g, the hydroxyl content reaches a maximum of 11.66 mmol/g. This observation can be attributed to the fact that a higher concentration of NMDG leads to a corresponding rise in the number of hydroxyl groups introduced onto the surface of PP-g-GMA fibers. However, as the availability of epoxy groups on the fiber surface becomes limited, the concentration of NMDG approaches a saturation point. An excessive concentration of NMDG may result in a reaction imbalance, potentially leading to incomplete reactions, the aggregation of NMDG, or the generation of by-products. Consequently, this may diminish the number of active sites and negatively impact the effectiveness of the ring-opening amination process.

### 3.5. Optimization of the Preparation Conditions for Imprinted Fibers

#### 3.5.1. The Influence of Different Crosslinking Agents on Adsorption Efficiency

**Figure 14:**
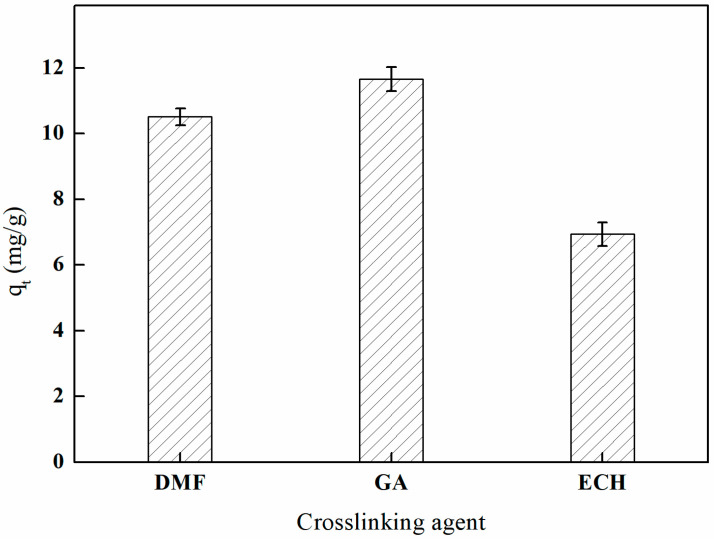
Effect of the crosslinking agent on adsorption (temperature of 60 °C and reaction duration of 3 h).

As illustrated in Figure 14, the adsorption effects of the various crosslinking agents on the imprinted fibers exhibited a distinct order: glutaraldehyde > *N*,*N*-dimethylformamide > epichlorohydrin. This variation in adsorption efficiency may be attributed to the presence of two aldehyde groups in glutaraldehyde, which facilitate rapid crosslinking with the hydroxyl groups on the surface of the PP-g-GMA-NMDG fibers. Such interactions not only enhance the coordination capacity with boron by introducing new active groups on the fiber surface but also contribute to the formation of a uniform network within the crosslinked material. This network preserves the porous structure of the fibers, thereby promoting boron adsorption. Following the application of glutaraldehyde crosslinking, the final imprinted fibers exhibit a boron adsorption capacity of 11.66 mg/g.

#### 3.5.2. The Influence of Different Crosslinking Temperatures on Adsorption Efficiency

**Figure 15:**
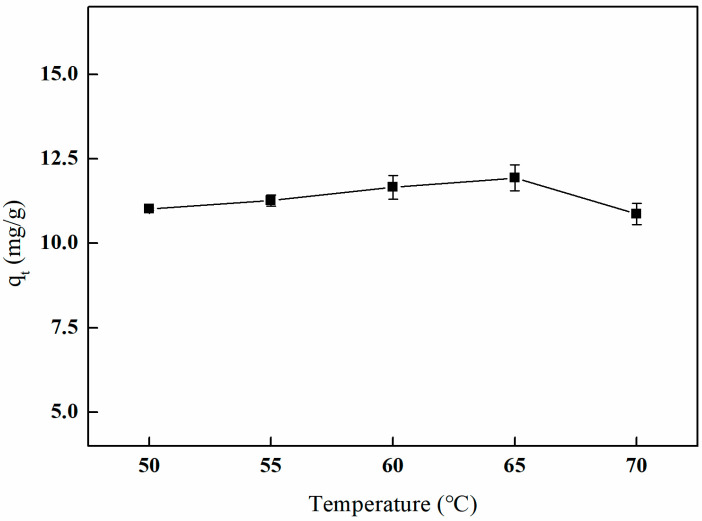
Effect of the crosslinking temperature on adsorption (5 mL of GA and reaction duration of 3 h).

As illustrated in Figure 15, the adsorption capacity of boron by the imprinted fibers initially increases with rising temperature, followed by a subsequent decline. The material exhibits the highest adsorption capacity for boron, quantified at 11.93 mg/g, at a crosslinking temperature of 65 °C. This phenomenon can be attributed to the fact that, within a certain temperature range, higher temperatures facilitate crosslinking reactions and enhance the internal crosslinking density of the material. However, excessively high temperatures may result in over-crosslinking, which can render the fiber material brittle and compromise the pore structure, ultimately affecting the adsorption performance of the imprinted fiber materials on boron.

#### 3.5.3. The Effect of Different Crosslinking Doses on Adsorption Efficiency

**Figure 16:**
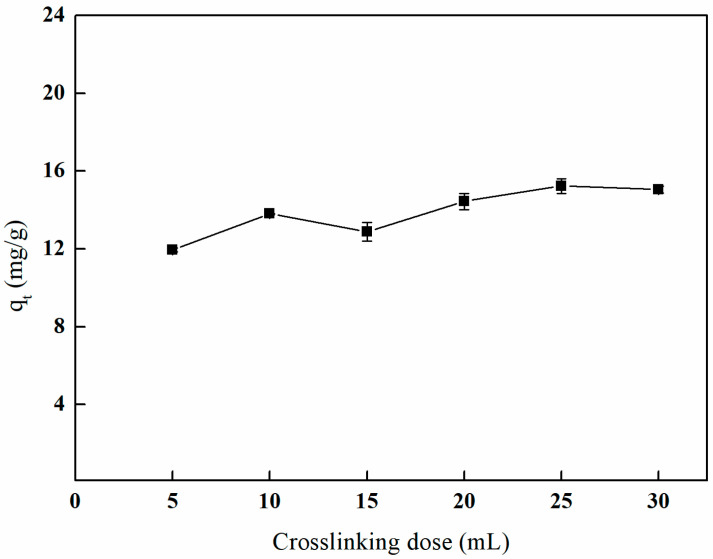
Effect of the crosslinking dose on adsorption (5 mL of GA, temperature of 65 °C and reaction duration of 3 h).

As illustrated in Figure 16, the adsorption capacity of boron by the imprinted fibers exhibits a gradual increase in response to the crosslinking dose, ultimately reaching a point of stabilization. Specifically, when the crosslinking dose is increased to 25 mL, the adsorption capacity of boron on the imprinted fibers attains its maximum value of 15.23 mg/g. This behavior can be attributed to the direct relationship between the crosslinking dose and the crosslinking density of the fiber material, thereby affecting the pore structure and the availability of active sites within the material. An insufficient crosslinking dose may result in an unstable crosslinking structure, ultimately compromising the adsorption capacity of boron by the imprinted fibers.

#### 3.5.4. The Influence of Different Crosslinking Times on Adsorption Efficiency

**Figure 17:**
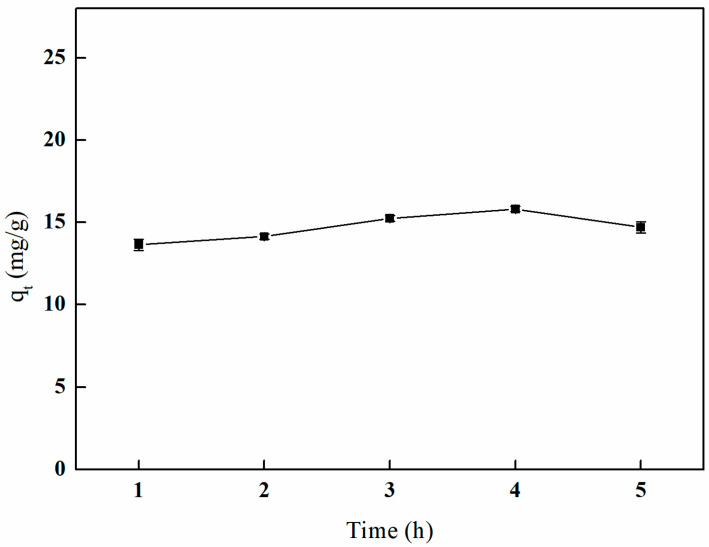
Effect of the crosslinking time on adsorption (25 mL of GA and temperature of 65 °C).

As illustrated in Figure 17, the adsorption capacity of boron by the imprinted fibers gradually increases with extended crosslinking time. The maximum adsorption capacity of 15.80 mg/g is achieved after 4 h of crosslinking. Subsequent to this duration, a decline in adsorption capacity is observed; however, it generally remains at a high level.

### 3.6. Characterization

#### 3.6.1. FT-IR

**Figure 18:**
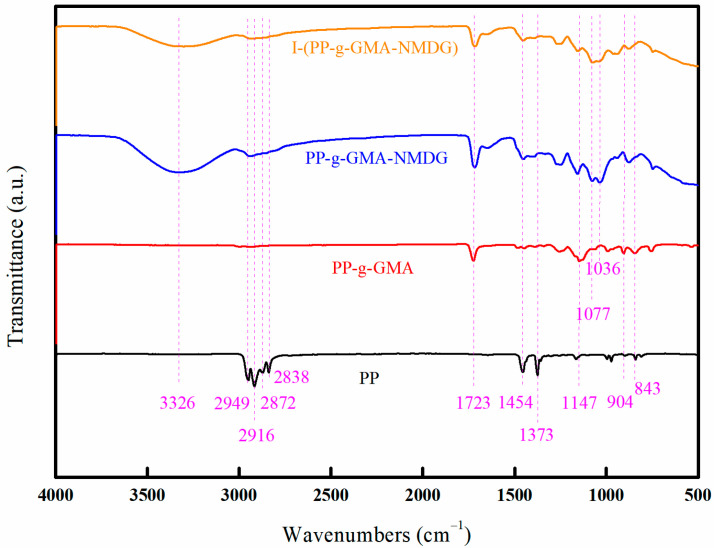
FT-IR images of PP, PP-g-GMA, PP-g-GMA-NMDG, and I-(PP-g-GMA-NMDG) fibers.

Figure 18 illustrates the infrared spectra of PP, PP-g-GMA, PP-g-GMA-NMDG, and I-(PP-g-GMA-NMDG) fibers. The spectra reveal that the characteristic peaks of PP fibers are observed at 2838 cm^−1^ and 2916 cm^−1^, corresponding to the symmetric and asymmetric stretching vibrations of the CH_2_ group, respectively [28,29]. Additionally, the peaks at 2872 cm^−1^ and 2949 cm^−1^ are associated with the symmetric and asymmetric stretching vibrations of the CH_3_ group. The peak at 1373 cm^−1^ is attributed to the bending vibration of CH_3_, while the peak at 1454 cm^−1^ corresponds to the bending vibration of CH_2_. In contrast, the PP-g-GMA fibers exhibit a characteristic peak at 1723 cm^−1^, which is associated with the stretching vibration absorption peak of the C=O group, and at 1147 cm^−1^, which represents the stretching vibration absorption peak of the C-O-C group [30]. The absorption peaks observed at 904 cm^−1^ and 843 cm^−1^ correspond to the stretching vibration absorption peaks of epoxy groups. As indicated by the Chemistry WebBook, the characteristic infrared absorption peak for C=C in GMA is typically found within the range of 1640–1560 cm^−1^; however, the infrared spectrum of PP-g-GMA fibers does not exhibit any peaks within this range. This absence suggests that GMA has been successfully grafted onto the surface of PP fibers through the cleavage of carbon–carbon double bonds. Compared to the infrared spectrum of PP-g-GMA fibers, the infrared spectrum of PP-g-GMA-NMDG fibers exhibits an O-H stretching vibration absorption peak at 3326 cm^−1^, a C-O stretching vibration absorption peak at 1077 cm^−1^, and a C-N stretching vibration absorption peak at 1036 cm^−1^. Meanwhile, the original characteristic peaks of the epoxy groups at 904 cm^−1^ and 843 cm^−1^ in PP-g-GMA fibers have been eliminated, indicating that the epoxy groups have undergone ring-opening reaction and subsequently interacted with the amine groups on NMDG, thereby successfully introducing NMDG onto the surface of PP-g-GMA fibers. After the surface ion imprinting reaction, the infrared spectrum of I-(PP-g-GMA-NMDG) fibers exhibits a retention of the characteristic absorption peaks observed in PP-g-GMA-NMDG fibers. The O-H stretching vibration absorption peak at 3326 cm^−1^, the C-O stretching vibration absorption peak at 1077 cm^−1^, and the C-N stretching vibration absorption peak at 1036 cm^−1^ are observed. These findings indicate the crosslinking reaction did not alter the active groups on the surface of the original PP-g-GMA-NMDG chelating fibers.

#### 3.6.2. XRD

**Figure 19:**
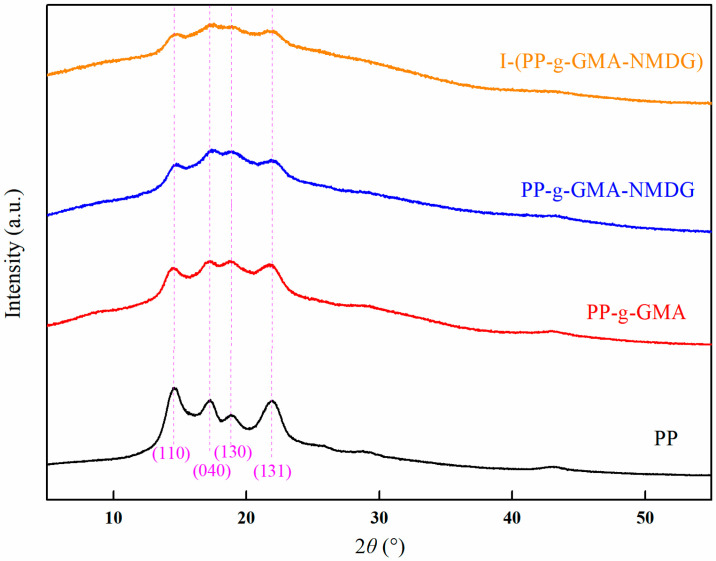
XRD patterns of PP, PP-g-GMA, PP-g-GMA-NMDG and I-(PP-g-GMA-NMDG) fibers.

Figure 19 illustrates the X-ray diffraction (XRD) patterns of PP, PP-g-GMA, PP-g-GMA-NMDG, and I-(PP-g-GMA-NMDG) fibers. The figure reveals that the characteristic diffraction peaks of PP fibers are observed at 2*θ* = 14°, 17°, 18°, and 21°, corresponding to the crystal planes (110), (040), (130), and (131), which are typical of the α crystalline form. Following the grafting of GMA via low-temperature plasma polymerization, the positions of the characteristic diffraction peaks of PP-g-GMA fibers remained consistent, indicating that the grafted fibers preserved the original crystalline structure of the PP fibers. Additionally, subsequent ring-opening amination did not reveal the emergence of new crystalline phases, thereby confirming that the chemical grafting reaction primarily occurred on the fiber surface and did not penetrate into the crystalline region. After the surface ion imprinting reaction, the I-(PP-g-GMA-NMDG) fibers exhibited the same characteristic diffraction peaks as the PP-g-GMA-NMDG fibers, suggesting that the crosslinking reaction was merely a straightforward bonding process that did not alter the internal crystalline structure of the imprinted fibers.

#### 3.6.3. TGA

**Figure 20:**
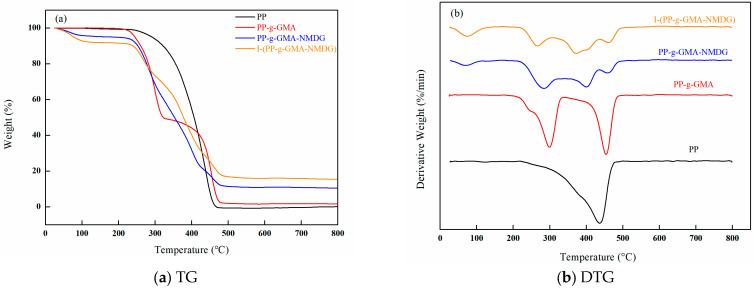
TGA spectra of PP, PP-g-GMA, PP-g-GMA-NMDG and I-(PP-g-GMA-NMDG) fibers. (**a**) TG; (**b**) DTG.

Figure 20 presents the thermogravimetric analysis (TGA) spectra for PP, PP-g-GMA, PP-g-GMA-NMDG, and I-(PP-g-GMA-NMDG) fibers. The data indicate that the original PP fibers experience a single weight loss stage, with the maximum rate of weight loss occurring at approximately 436 °C. In contrast, the PP-g-GMA fibers display two distinct weight loss stages: one occurring at a temperature corresponding to the peak weight loss rate of the original PP fibers, and another rapid weight loss stage occurring at around 299 °C. This latter phase of weight loss is likely attributable to the degradation of the grafted GMA onto the surface of the PP fibers. The PP-g-GMA-NMDG fibers exhibit three distinct stages of weight loss, two of which correspond to the rapid weight loss temperatures observed in the PP-g-GMA fibers. The third rapid weight loss temperature occurs around at 400 °C, which can be attributed to the degradation of NMDG introduced onto the surface of the PP-g-GMA fibers via the ring-opening amination reaction. In comparison, the I-(PP-g-GMA-NMDG) fibers also display three similar weight loss stages; however, throughout the entire temperature range, the mass loss of the I-(PP-g-GMA-NMDG) fibers is the lowest. Additionally, their thermal decomposition temperature is slightly higher than that of the PP-g-GMA-NMDG fibers, indicating enhanced thermal stability, likely due to the effects of the crosslinking. Overall, it can be concluded that modifications through plasma graft polymerization, ring-opening amination reactions, or ion imprinting reactions have not adversely affected internal macromolecular structure of PP, and the modified materials continue to exhibit good thermal stability.

#### 3.6.4. SEM

**Figure 21:**
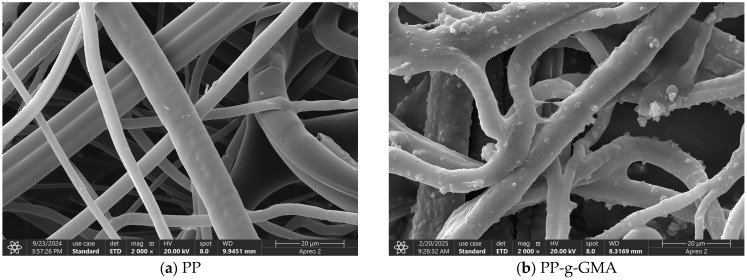

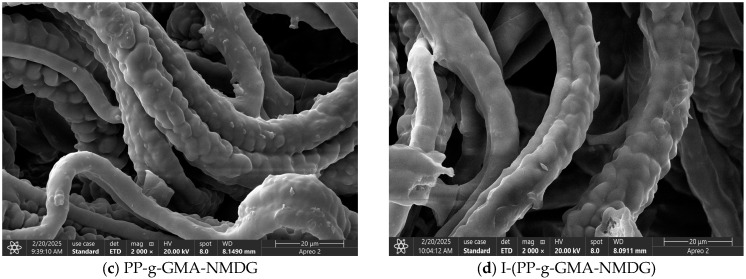
SEM images of PP, PP-g-GMA, PP-g-GMA-NMDG and I-(PP-g-GMA-NMDG) fibers.

Figure 21 illustrates the scanning electron microscopy (SEM) images of PP, PP-g-GMA, PP-g-GMA-NMDG, and I-(PP-g-GMA-NMDG) fibers. In image (a), the surface of PP is characterized by a smooth texture, and the overall fibers exhibit a fluffy appearance. Following the plasma graft polymerization of GMA, image (b) reveals that the surface of the PP-g-GMA fibers is marked by the presence of particulate matter, resulting in a roughened texture. These particles are likely a consequence of the graft polymerization of GMA on the fiber surface. After subsequent ring-opening amination, image (c) indicates that the particles previously observed on the surface of the PP-g-GMA fibers have been removed, thereby restoring the smoothness of the fiber surface. Compared to the surface of PP-g-GMA fibers, the surface of PP-g-GMA-NMDG fibers appears more multi-layered and denser, while the overall fiber structure is less fluffy than that of the PP material. This alteration is attributed to the amination of GMA and NMDG, which altered the original surface structure and resulted in the formation of new substances. In contrast to PP-g-GMA-NMDG fibers, the surface of I-(PP-g-GMA-NMDG) fibers is characterized by a rough texture and is densely packed with small pores that have accumulated on the original chelated fiber surface. This phenomenon may be due to elution resulting from the crosslinking reaction, which is consistent with findings reported in the literature [31,32,33].

#### 3.6.5. EDS

**Figure 22:**
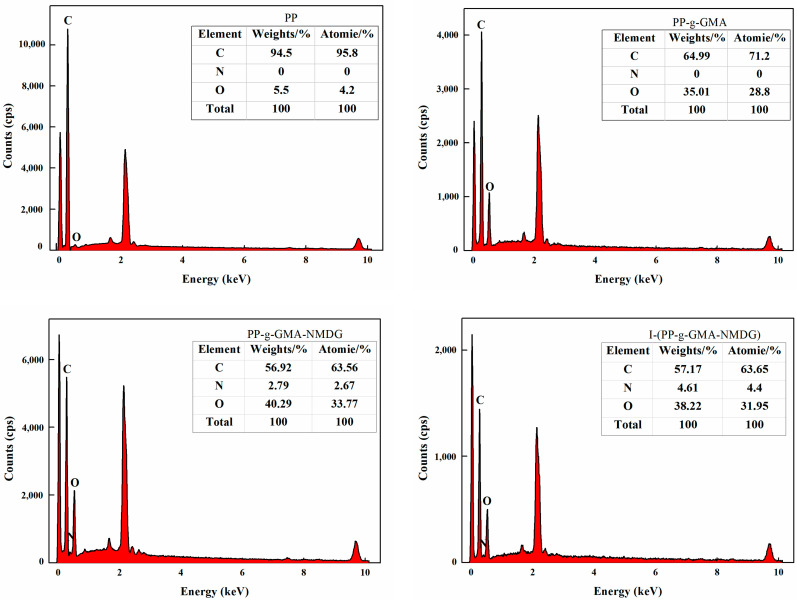
EDS images of PP, PP-g-GMA, PP-g-GMA-NMDG and I-(PP-g-GMA-NMDG) fibers.

Figure 22 presents the Energy Dispersive Spectroscopy (EDS) spectra for PP, PP-g-GMA, PP-g-GMA-NMDG, and I-(PP-g-GMA-NMDG) fibers. The figure illustrates the elements and their respective concentrations in different materials at various stages of the reaction. The PP fibers are characterized by a high carbon (C) content due to their inherent structure, while the low oxygen (O) levels observed in the spectrum can be attributed to the reaction with atmospheric oxygen during the preparation of the PP melt-blown fibers. As the reaction progresses, a gradual reduction in the carbon content is noted in the PP-g-GMA fibers, accompanied by a consistent increase in the oxygen content. This change is indicative of the significant grafting of GMA onto the surface of the PP fibers, as the epoxy groups present in GMA contribute a significant amount of oxygen (O), thereby elevating the overall oxygen content in the PP-g-GMA fibers. Following the ring-opening amination reaction, a further decline in the carbon (C) content is observed in the PP-g-GMA-NMDG fibers, alongside an increase in the oxygen content and the introduction of nitrogen (N). These are due to NMDG, which acts as an amination reagent and successfully grafts onto the surface of the PP-g-GMA fibers. NMDG is rich in hydroxyl (OH) and amino (NH_2_) groups, which further enhance the oxygen content. These findings substantiate the successful modification of the PP-g-GMA-NMDG fibers. The elemental composition of each fiber material, both before and after the crosslinking reaction, indicates increases in the contents of carbon (C) and nitrogen (N) compared to their levels prior to crosslinking, while the oxygen (O) content has decreased. This alteration may be attributed to the interaction of the crosslinking agent glutaraldehyde with the PP-g-GMA-NMDG chelated fibers, resulting in minor changes to the overall elemental composition on the surface of the I-(PP-g-GMA-NMDG) fibers.

#### 3.6.6. WCA

**Figure 23:**
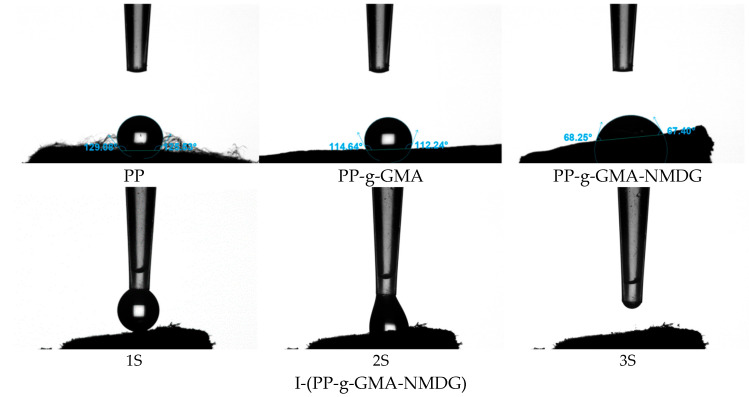
Water contact angle images of PP, PP-g-GMA, PP-g-GMA-NMDG and I-(PP-g-GMA-NMDG) fibers.

Figure 23 presents the water contact angle (WCA) test results for PP, PP-g-GMA, PP-g-GMA-NMDG, and I-(PP-g-GMA-NMDG) fibers. The experimental data reveal that the WCA of the original PP fibers is 129°, which is characteristic of hydrophobic materials. This hydrophobicity can be attributed to their non-polar molecular chain structure (-CH_2_-CH_2_-) that lacks hydrophilic functional groups. Following plasma graft polymerization treatment, the water contact angle of the PP-g-GMA fibers decreases to 114°, indicating a reduction in hydrophobicity, although these fibers remain hydrophobic. As the ring-opening amination reaction progresses, the WCA of the PP-g-GMA-NMDG fibers significantly decreases to 68°, demonstrating an enhancement in the wettability of the fiber surface. This improvement can be attributed to the introduction of a significant number of hydrophilic hydroxyl (OH) groups from NMDG, which enhances the overall hydrophilicity of the PP-g-GMA-NMDG fibers. As the ion imprinting reaction progresses, the resulting I-(PP-g-GMA-NMDG) imprinted fibers exhibit a phenomenon where the contact angle cannot be directly measured using an optical contact angle measuring instrument as deionized water spreads immediately upon contact with the fiber surface, indicating effective wetting. This observation indicates that the hydrophilicity of the crosslinked imprinted fiber material is further enhanced, thereby facilitating the adsorption of boron in aqueous solutions.

### 3.7. Exploration of Adsorption Performance

#### 3.7.1. Adsorption Experiment with Different Solution pH Values

**Figure 24:**
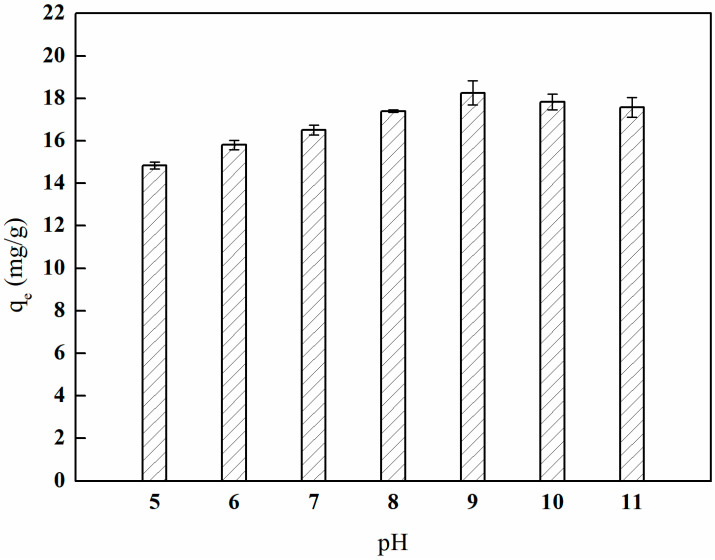
Effect of pH on boron adsorption by the imprinted fibers.

Figure 24 illustrates that as the pH value continuously increases, the adsorption capacity of I-(PP-g-GMA-NMDG) ion-imprinted fibers reaches its peak at pH 9. The I-(PP-g-GMA-NMDG) ion-imprinted fibers also exhibit some degree of adsorption capacity at pH values below 7. This may be attributed to the presence of specific boronic acid adsorption sites on the surface of the boronic acid ion-imprinted polymers, as well as some non-specific adsorption sites. These sites interact with boronic acid molecules through van der Waals forces and other interactions, thereby facilitating the adsorption of boronic acid onto their surfaces. As the pH value increases, the concentration of B(OH)_4_^−^ in the solution also rises, leading to an increase in the corresponding adsorption capacity of borate ions. At pH 9, the adsorption capacity of boron by the imprinted fibers reaches its highest value, quantified at 18.24 mg/g [34,35]. Subsequently, the adsorption capacity of boron gradually decreases, likely due to the increased concentration of OH⁻ ions in the solution, which enhances repulsion with B(OH)_4_^−^ and negatively impacts the adsorption capacity of boron.

#### 3.7.2. Adsorption Kinetics Experiment

**Figure 25:**
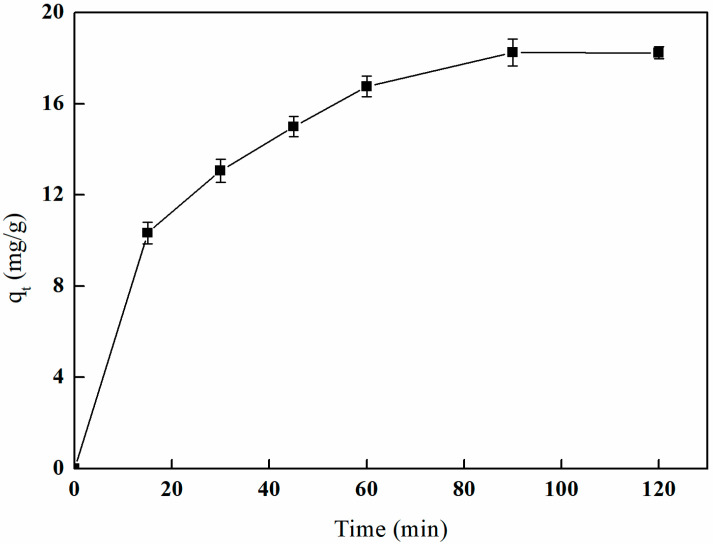
Effect of time on boron adsorption by I-(PP-g-GMA-NMDG) fibers.

Figure 25 illustrates the effect of I-(PP-g-GMA-NMDG) imprinted fibers on boron adsorption in solution across different time intervals. The graph indicates that the adsorption rate is highest during the initial 15 min, after which it begins to decline. By the time the adsorption period reaches 60 min, the amount of boron adsorbed exceeds 90% of the saturated adsorption capacity. At 90 min, the adsorption amount stabilizes and approaches equilibrium, ultimately reaching a saturated adsorption capacity of 18.26 mg/g.

Meanwhile, the investigation into the effect of N-(PP-g-GMA-NMDG) non-imprinted fibers on boron adsorption in solution over varying time intervals reveals that these non-imprinted fibers require 120 min to achieve adsorption equilibrium. In contrast, I-(PP-g-GMA-NMDG) imprinted fibers possess three-dimensional cavities that are precisely aligned with boron in terms of spatial dimensions, charge, and morphology, leading to a more rapid adsorption rate.

In order to gain a deeper understanding of the adsorption characteristics of I-(PP-g-GMA-NMDG) imprinted fibers for boron in solution, this study analyzes and models the adsorption process of boron using both pseudo-first-order and pseudo-second-order kinetic models to investigate its adsorption behavior.

(1) Pseudo-first-order kinetic model

(4) $$\log\left(q_{e}-q_{t}\right)=logq_{e}-k_{1}t$$

where *q*_e_ represents the equilibrium adsorption amount, mg/g; *q*_t_ denotes the adsorption amount at time *t*, mg/g; k_1_ is the rate constant of the pseudo-first-order kinetic model, 1/min; and *t* indicates the adsorption time, min.

(2) Pseudo-second-order kinetic model

(5) $$\frac{t}{q_{t}}=\frac{1}{k_{2}{q_{e}}^{2}}+\frac{t}{q_{e}}$$

where *q*_t_ represents the adsorption amount at time *t*, mg/g; *q*_e_ denotes the equilibrium adsorption amount, mg/g; k_2_ is the rate constant of the pseudo-second-order kinetic model, g/mg·min; and *t* indicates the adsorption time, minutes.

**Figure 26:**
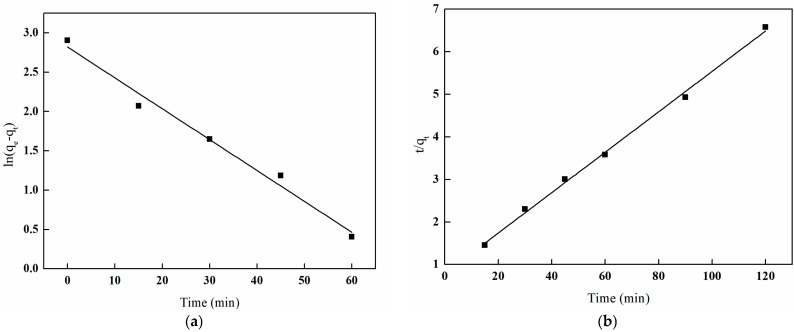
Plots of pseudo-first-order and pseudo-second-order models of I-(PP-g-GMA-NMDG) fibers. (**a**) Pseudo-first-order model; (**b**) pseudo-second-order model.

Figure 26 illustrates the kinetic fitting equation for boron adsorption in solution using I-(PP-g-GMA-NMDG) imprinted fibers, while Table 4 presents the kinetic parameters associated with this process. An analysis of the figure and table reveals that the correlation coefficient (*R*^2^) for the pseudo-first-order kinetic model is 0.9798, in contrast to a correlation coefficient (*R*^2^) of 0.9968 for the pseudo-second-order kinetic model. The latter coefficient is closer to the theoretical maximum of one, and the theoretical saturation adsorption capacity aligns more closely with the experimental results. According to classical adsorption theory, the pseudo-first-order model posits that the adsorption process is governed by physical diffusion mechanisms, such as pore diffusion or film diffusion. In contrast, the pseudo-second-order model is based on a chemical adsorption mechanism, which involves electron transfer and chemical bonding between the adsorbent and the adsorbate [36].

Therefore, based on the relevant data from the charts, the I-(PP-g-GMA-NMDG) imprinted fibers demonstrate that the kinetic behavior for boron adsorption in solution is more accurately represented by the pseudo-second-order kinetic model, which is a critical factor influencing the adsorption rate.

In the field of engineering, the semi-saturated adsorption time (t_1/2_) is usually used to describe the adsorption rate of adsorbent materials. Therefore, by substituting the semi-saturated adsorption capacity and corresponding time t_1/2_ into the pseudo-second-order kinetic equation, the relationship t_1/2_ = 1/k_2_q_e_ can be derived. Then, the t_1/2_ of I-(PP-g-GMA-NMDG) imprinted fibers is obtained by fitting the parameters of the pseudo-second-order kinetic equation, yielding a value of 16.4 min. This indicates that I-(PP-g-GMA-NMDG) imprinted fibers can reach half of the saturated adsorption capacity in a relatively short time.

#### 3.7.3. Adsorption Isotherm

Adsorption isotherms are utilized to describe the relationship between the amount of adsorbate retained by an adsorbent at equilibrium at a specific temperature and the corresponding concentrations of the solute. This analysis is essential for evaluating the adsorption performance of the adsorbent. In this study, the Langmuir, Freundlich, and Temkin adsorption isotherm models were employed to analyze and fit the data regarding boron adsorption by I-(PP-g-GMA-NMDG) imprinted fibers.

(1) Langmuir adsorption isotherm model

(6) $$q_{e}=\frac{q_{max}K_{L}C_{e}}{1+K_{L}C_{e}}$$

where *q*_e_ represents the equilibrium adsorption amount, mg/g; *q*_max_ denotes the theoretical maximum adsorption amount, mg/g; K_L_ is the Langmuir adsorption equilibrium constant, L/mg; and *C*_e_ indicates the equilibrium concentration of the solution, mg/L.

(2) Freundlich adsorption isotherm model

(7) qe=KFCe1n

The linear form is

(8) $$logq_{e}=logK_{F}+\frac{1}{n}logC_{e}$$

where *q*_e_ represents the equilibrium adsorption amount, mg/g; K_F_ and 1/*n* are the Freundlich constants; and *C*_e_ denotes the equilibrium concentration of the solution, mg/L.

(3) Temkin adsorption isotherm model

(9) $$q_{e}=B_{T}\ln\left(K_{T}C_{e}\right)$$

where *q*_e_ represents the equilibrium adsorption amount, mg/g; *B*_T_ denotes the slope of the line; K_T_ is the Temkin adsorption equilibrium constant; and *C*_e_ indicates the equilibrium concentration of the solution, mg/L.

**Figure 27:**
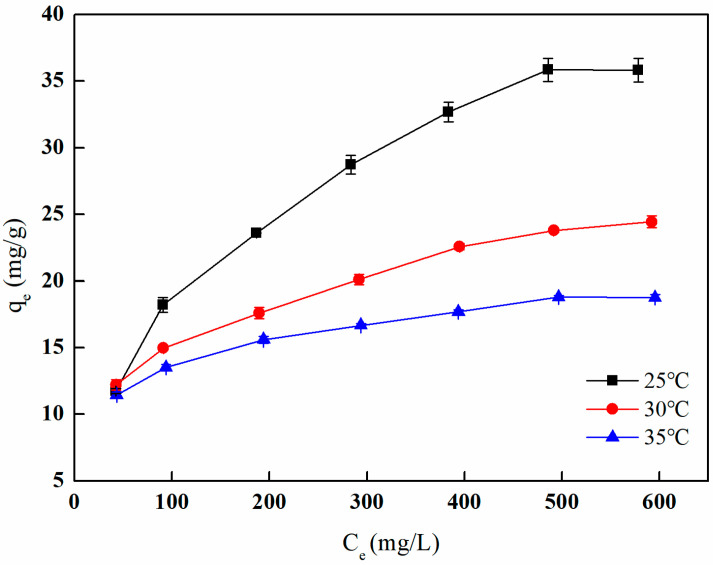
Adsorption isotherm of boron adsorption by I-(PP-g-GMA-NMDG) fibers.

Figure 27 illustrates the relationship between the boron adsorption capacity of I-(PP-g-GMA-NMDG) imprinted fibers at various temperatures and different boron equilibrium concentrations. The figure demonstrates that an increase in the boron equilibrium concentration corresponds with a rise in the boron adsorption capacity. This trend can be attributed to the fact that elevated concentrations promote more frequent collisions between the functional groups on the fiber surface and the adsorbate in the solution, thereby enhancing boron adsorption. As the active sites on the fiber surface become increasingly occupied, the adsorption capacity eventually approaches a state of saturation, with the maximum saturated adsorption capacity recorded at 35.85 mg/g at 25 °C.

**Figure 28:**
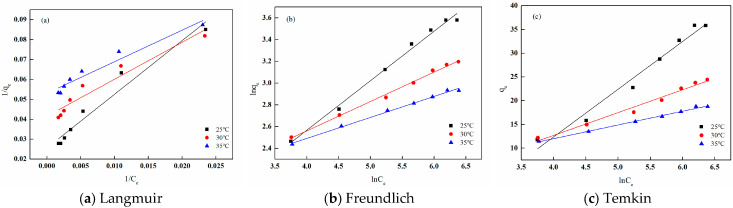
Fitting of the adsorption isotherms of I-(PP-g-GMA-NMDG) fibers at different temperatures.

The adsorption behavior of I-(PP-g-GMA-NMDG) imprinted fibers was evaluated through the application of three adsorption isotherm models: Langmuir, Freundlich, and Temkin. The fitting results and related parameters are illustrated in Figure 28 and Table 5, respectively. From the fitting curves and the parameter table, it is evident that the Freundlich adsorption isotherm model exhibits the highest fitting correlation coefficient at various temperatures, all exceeding 0.99. This suggests that the adsorbent material possesses a heterogeneous adsorption surface characterized by diverse adsorption energies for the adsorbate, and that it facilitates multilayer adsorption. Furthermore, the Freundlich adsorption isotherm model includes a significant parameter, 1/n, which, when calculated, consistently yields values less than 0.5 at different temperatures. This finding indicates that I-(PP-g-GMA-NMDG) imprinted fibers have a strong capacity to adsorb boron [37].

In this experiment, the maximum adsorption capacity of I-(PP-g-GMA-NMDG) imprinted fibers for boron was determined to be 35.85 mg/g. A review of recent studies on boron adsorption materials indicates that the boron adsorbent developed in this study demonstrates a significant advantage in terms of adsorption capacity compared to other adsorbents of a similar nature, as illustrated in Table 6. Simultaneously, it has been observed that the majority of current research on boron adsorbents relies on the principle of boron affinity. As a result, the selection of adsorbents in complex environments is often compromised due to the interference of competitive ions, necessitating further treatment of the adsorbent in subsequent stages. This study introduces a novel boron adsorption material that integrates ion imprinting technology with the principle of boron affinity, thereby addressing these challenges and offering a new perspective on boron adsorption methodologies.

#### 3.7.4. Adsorption Thermodynamics Experiment

Aqueous boric acid solutions with concentrations of 50, 100, 200, 300, 400, 500, and 600 mg/L were prepared to simulate water samples. The pH of the solutions was adjusted to the optimal conditions, followed by the addition of 10 mL of the boric acid solution. Subsequently, 0.01 g of the I-(PP-g-GMA-NMDG) fiber material was placed in a stoppered glass sample bottle. The samples were agitated at a speed of 200 r/min in a constant-temperature water bath at 25 °C, 30 °C, or 35 °C for 2 h. The concentrations of boron in the aqueous solutions were quantified before and after the adsorption process by filtering the solutions.

In order to enhance the comprehension of the thermodynamic properties associated with the boron adsorption process using I-(PP-g-GMA-NMDG) imprinted fibers, this study employs the Gibbs free energy equation to elucidate the energy transformations that occur during adsorption. This study meticulously examines parameters including the change in Gibbs free energy (∆G^0^), the change in enthalpy (∆H^0^), and the change in entropy (∆S^0^) to provide a comprehensive assessment of the material’s adsorption efficacy.

(10) $$∆G^{0}=-RTlnK_{0}$$

(11) $$lnK_{0}=-\frac{∆H^{0}}{RT}+\frac{∆S^{0}}{R}$$

where K_0_ represents the thermodynamic equilibrium constant; *T* denotes the absolute temperature measured in Kelvin, K; and R is the ideal gas constant, valued at 8.314 J/(mol·K).

**Figure 29:**
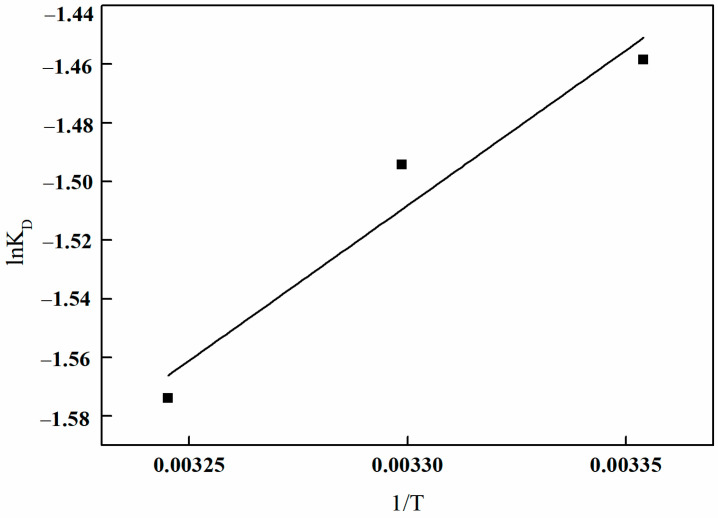
Plots of lnK_0_ and 1/*T* of I-(PP-g-GMA-NMDG) fibers.

A linear regression analysis was conducted to plot lnK_0_ against 1/*T*, as illustrated in Figure 29. The graph demonstrates a strong linear correlation between lnK_0_ and 1/*T*. The slope and intercept of the fitted linear equation can be determined. Utilizing Equations (9) and (10), the relevant data for the thermodynamic parameters can be calculated, as presented in Table 7.

Table 7 demonstrates that the ∆G^0^ values for boron adsorption onto I-(PP-g-GMA-NMDG) imprinted fibers at various temperatures are all positive, indicating that the adsorption process is non-spontaneous from a thermodynamic perspective. The ∆H^0^ values are consistently negative, suggesting that the adsorption process is exothermic, and an increase in temperature is unfavorable for adsorption. This phenomenon is further illustrated in Figure 27, which illustrates a decline in the amount of adsorption with rising temperature. The ∆S^0^ values are also negative, indicating that the adsorption reaction results in a gradual decrease in disorder of the adsorbate, leading to a more ordered adsorption system. This phenomenon occurs because the adsorbate transitions from a state of free movement to being bound to the surface of the adsorbent, thereby diminishing its degrees of freedom and contributing to a decrease in the overall disorder of the system.

#### 3.7.5. Adsorption Selectivity Experiment

Based on the ionic composition of the salt lake brine, simulated competitive solutions were prepared using boric acid, anhydrous sodium carbonate, potassium bicarbonate, magnesium sulfate, anhydrous calcium chloride, barium chloride, and heptahydrate zinc sulfate. The initial concentration of boric acid was set at 100 mg/L, while the concentrations of the competing ions Na^+^, K^+^, Ca^2+^, Mg^2+^, SO_4_^2−^, and Cl^−^ were 20, 40, 60, 80, and 100 mg/L, respectively. This configuration resulted in the formation of six groups of ion competition systems: B(OH)_4_^−^ + Na^+^, B(OH)_4_^−^ + K^+^, B(OH)_4_^−^ + Ca^2+^, B(OH)_4_^−^ + Mg^2+^, B(OH)_4_^−^ + SO_4_^2−^, and B(OH)_4_^−^ + Cl^−^. A 10 mL sample of the simulated competitive solution was taken, and 0.01 g of the I-(PP-g-GMA-NMDG) fiber material was placed in a stoppered glass sample bottle. The bottle was shaken in a constant temperature water bath at 25 °C at a speed of 200 rpm for 2 h, after which the concentration of boron in the aqueous solution was measured. The distribution coefficient *k*_d_, selectivity coefficient (*k*), and relative selectivity coefficient (*k*′) were calculated using the following formulas [45]:

(12) $$k_{d}=\frac{C_{0}-C_{e}}{C_{e}}\times \frac{V}{m}$$

(13) $$k=\frac{k_{d\left(B\right)}}{k_{d\left(M\right)}}$$

(14) $$k^{\prime }=\frac{k_{IIP}}{k_{NIP}}$$

where *k*_d_ represents the distribution coefficient (L/g); *k* denotes the selectivity coefficient; *k*′ indicates the relative selectivity coefficient; M refers to the competing ion; IIP stands for the imprinted fiber; and NIP signifies the non-imprinted fiber.

**Figure 30:**
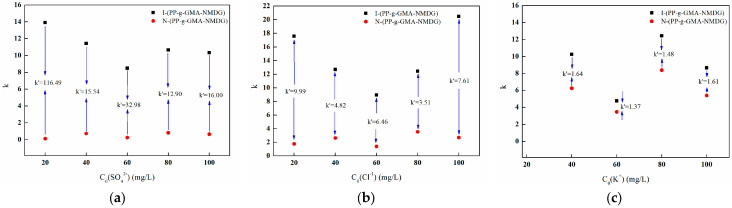
*k* and *k*′ values for different concentrations of ions. (**a**) SO_4_^2−^; (**b**) Cl^−^; and (**c**) K^+^.

Figure 30a,b illustrate the *k* values and *k*′ values of the competitive ions SO_4_^2−^ and Cl^−^ at various concentrations for both imprinted and non-imprinted fibers. The data presented in the figures demonstrate that the presence of competitive anions at different concentrations alters the *k* values for both types of fibers. This indicates that competitive ions significantly influence the adsorption process of boron. Moreover, the *k* value of the imprinted fibers is greater than that of the non-imprinted fibers, resulting in a notable increase in the *k*′ value. This phenomenon suggests that the specific recognition sites on the surface of the imprinted fibers possess a high affinity for the target ions during the adsorption process. In contrast, the non-imprinted fibers lack these specific recognition sites, which leads to inadequate selective adsorption performance. Additionally, at the same concentration, *k*′ for the competitive ion Cl^−^ is lower than that for SO_4_^2−^, indicating that the adsorption competitiveness follows the order Cl^−^ > SO_4_^2−^.

Figure 30c illustrates the *k* values and *k*′ values of both imprinted and non-imprinted fibers with varying concentrations of the competing ion K^+^. The figure demonstrates that the presence of K^+^ at different concentrations alters the *k* value of both types of fibers, suggesting that K^+^ influences the adsorption process of boron. Additionally, the *k* value of the imprinted fibers is greater than that of the non-imprinted fibers, with the ratio remaining above one. This observation indicates that the specific recognition sites on the surface of the imprinted fibers exhibit a pronounced ability to recognize the target ions during the adsorption process.

In comparison to anions, the *k*′ value of the adsorption materials in the presence of cations is relatively low, indicating that cations have a limited impact on the selective adsorption of boron by the imprinted fibers. This finding is consistent with the adsorption phenomena noted in binary systems containing competing ions such as Na^+^, Mg^2+^, and Ca^2+^. Regardless of whether the materials are imprinted or non-imprinted, the adsorption capacity for Na^+^, Mg^2+^, and Ca^2+^ ions remains insignificant; however, these materials still demonstrate effective adsorption of boron.

In summary, the selectivity coefficients (*k*) for the imprinted fibers concerning B(OH)_4_^−^/SO_4_^2−^ and B(OH)_4_^−^/Cl^−^ are both significantly greater than one, indicating that the imprinted fibers exhibit a high selectivity for B(OH)_4_^−^. The relatively high selectivity coefficient (*k*′) suggests that the selectivity of the imprinted fibers surpasses that of the non-imprinted fibers. Consequently, the high selectivity of boron-imprinted fiber materials for boron can be attributed to the efficacy of the imprinting process, which creates imprinted cavities on the surface of the PP-g-GMA-NMDG chelating fibers. These cavities are precisely aligned with the spatial three-dimensional structure, shape, size, and charge of boron, thereby demonstrating a strong selectivity for the template ions.

### 3.8. Adsorption Mechanism of Borate Ions

The study of borate ion adsorption on I-(PP-g-GMA-NMDG) imprinted fibers across varying pH levels reveals the presence of both specific and non-specific adsorption sites From a geometric micro-area perspective, the borate ion-imprinted polymer exhibits a distinct three-dimensional spatial structure, where the imprinted cavities are precisely aligned with the size, charge, shape, and spatial configuration of the borate ions. This alignment facilitates rapid interaction with borate ions and promotes successful adsorption onto the surface of the imprinted fibers. In contrast, competing ions are unable to occupy the imprinted cavities due to differences in the ionic radius, shape, size, and charge compared to the template ions, which restricts their adsorption. The process of boron adsorption is predominantly governed by van der Waals forces, electrostatic forces, hydrogen bonding, hydrophobic interactions, and specific bonding interactions between the active functional groups on the adsorbent surface and the borate ions.

### 3.9. Investigation of Reuse Performance

**Figure 31:**
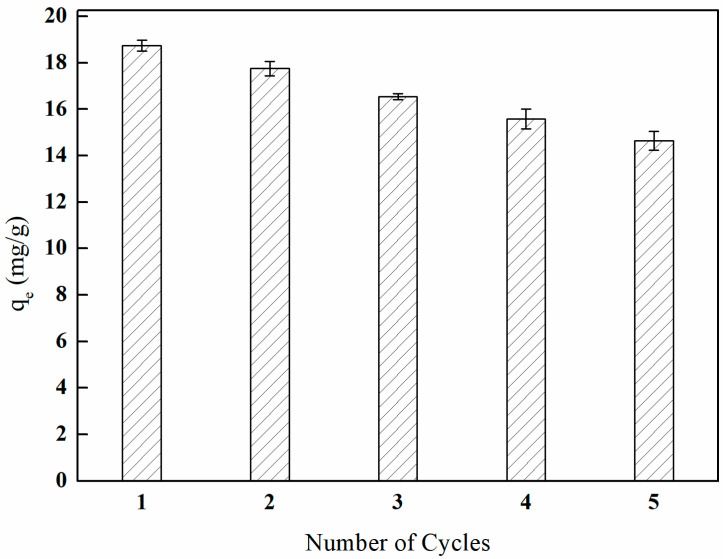
Regeneration performance of I-(PP-g-GMA-NMDG) fibers.

The regenerability of adsorbent materials is crucial for their overall performance. The regeneration performance of I-(PP-g-GMA-NMDG) imprinted fibers was examined through a series of adsorption–desorption cycling experiments. The results are presented in Figure 31. As the number of adsorption–desorption cycles increased, the adsorption efficiency of the I-(PP-g-GMA-NMDG) imprinted fibers gradually declined. After five cycles of adsorption, the material maintained 78.10% of its initial adsorption capacity.

### 3.10. Characterization of the Imprinted Fibers Before and After Adsorption/Desorption

This study examines the impact of borate ions on the regeneration and reuse of imprinted fibers by analyzing the modifications in surface functional groups of the fibers following prolonged use, utilizing Fourier Transform Infrared Spectroscopy (FT-IR). Additionally, this research evaluates alterations in surface morphology through electron microscopy techniques.

#### 3.10.1. FT-IR

**Figure 32:**
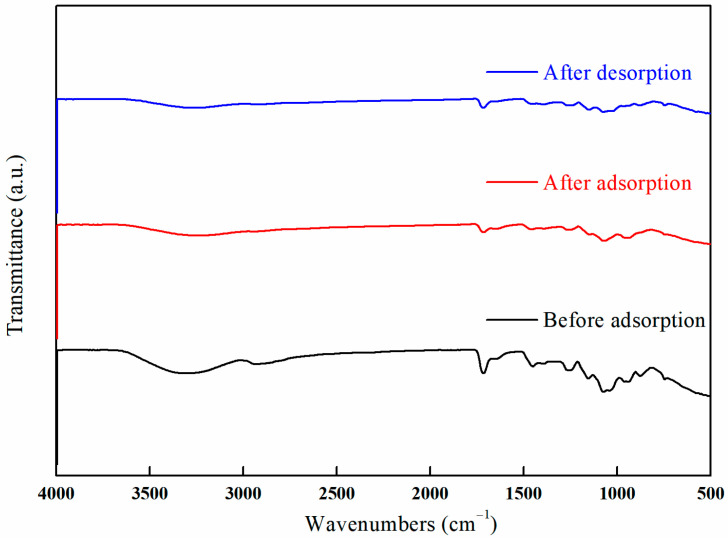
FT-IR spectra of the I-(PP-g-GMA-NMDG) fibers before and after adsorption/desorption.

Figure 32 illustrates the infrared spectra of I-(PP-g-GMA-NMDG) fibers before and after the adsorption/desorption of borate ions. The figure indicates that after the I-(PP-g-GMA-NMDG) imprinted fibers adsorb borate ions, the relevant characteristic peaks present before adsorption do not disappear; instead, the intensity of these absorption peaks is significantly reduced. This observation suggests that the hydroxyl groups have coordinated with the borate ions. Furthermore, a stretching vibration absorption peak corresponding to the B-O bond in borate esters is observed at 1390 cm^−1^, thereby confirming the successful adsorption of borate ions onto the surface of the I-(PP-g-GMA-NMDG) imprinted fibers.

A comparative analysis of the spectra obtained before and after adsorption process reveals a notable increase in the peak intensity at 1066 cm^−1^. This increase is attributed to the enhanced bending vibration of the borate hydroxyl group following boron adsorption. Conversely, a decrease in the intensity of the O-H stretching vibration peak at 3306 cm^−1^ is observed. Furthermore, an examination of the infrared spectra before and after desorption indicates that the imprinted fibers retain the characteristic peaks observed prior to desorption (after adsorption). It is particularly noteworthy that the reduction in the intensity of the O-H stretching vibration peak is more pronounced after desorption, indicating that borate ions have effectively substituted the O-H groups during the adsorption process.

The analysis of the data presented in Figure 31, which pertains to the regeneration performance of the imprinted fibers, indicates that the decrease in boron adsorption efficiency during subsequent continuous adsorption–desorption cycles suggests that the active sites on the surface of the imprinted fibers have not been completely eluted. This observation indicates that the boron associated with the imprinted fibers exhibits a strong resistance to desorption, while a certain proportion of boron is physically adsorbed and can be easily desorbed due to its weaker binding interactions. Furthermore, the intensity of the characteristic peaks after desorption process is markedly diminished in comparison to those observed prior to the adsorption phase.

#### 3.10.2. SEM

**Figure 33:**
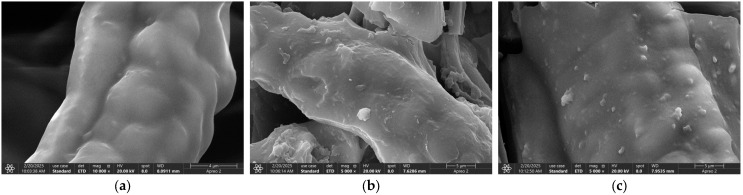
SEM images of I-(PP-g-GMA-NMDG) fibers before and after adsorption/desorption. (**a**) Before adsorption; (**b**) after adsorption; and (**c**) after desorption.

Figure 33 presents electron microscopy images of the I-(PP-g-GMA-NMDG) fibers before and after the adsorption/desorption process. Figure 33a,b illustrates that after the adsorption of borate ions onto the I-(PP-g-GMA-NMDG) imprinted fibers, the initially smooth surface exhibits increased roughness, accompanied by the presence of scattered flaky substances. These features may represent the morphological characteristics of borate ions adhering to the fiber surface. Subsequently, desorption was conducted, and the electron microscopy image of the imprinted fibers after desorption is displayed in Figure 33c. It is evident that a majority of the flaky borate ions that were initially adsorbed onto the fiber surface have been removed; however, some borate ions remain, and the rough fiber surface has reverted to a smoother texture. These observations are consistent with the infrared spectra and the results obtained from the performance evaluation.

## 4. Conclusions

In order to explore a method for preparing new ion-selective adsorption materials, GMA and NMDG were successfully grafted onto PP fibers through low-temperature plasma graft polymerization, open-loop amination, and ion imprinting reactions, and the parameters involved in the preparation process were systematically optimized. A series of relevant characterizations were conducted on the fiber materials before and after modification to verify the feasibility of the preparation. The hydrophilicity of I-(PP-g-GMA-NMDG) fibers was greatly improved, which was beneficial for treatment in aqueous solution. The adsorption process was determined to be non-spontaneous and exothermic, and was well fitted by pseudo-second-order kinetic and Freundlich isotherm models. The maximum adsorption capacity was 35.85 mg/g with boron concentration of 500 mg/L at pH = 9 and 25 °C. The synthesized I-(PP-g-GMA-NMDG) fibers exhibited a high adsorption capacity, commendable selectivity, consistent reproducibility, and ease of recovery, thereby demonstrating superior performance in comparison to other adsorbents. These results highlight the significant potential for practical applications in the treatment of wastewater containing boron.

## Figures and Tables

**Table 1:** Factor level and coding.

Factor	Code	Encoding Level		
		−1	0	1
GMA concentration/%	A	20	25	30
Temperature/°C	B	75	80	85
Time/h	C	1.5	2	2.5

**Table 2:** Experimental design and results.

Experiment Number	A	B	C	GP (%)
1	25	80	2	157.24
2	20	85	2	106.81
3	30	85	2	160.32
4	20	80	1.5	100.19
5	30	80	1.5	121.71
6	30	75	2	147.45
7	25	75	2.5	181.36
8	20	75	2	106.45
9	30	80	2.5	171.68
10	25	85	1.5	104.65
11	20	80	2.5	172.52
12	25	80	2	152.67
13	25	80	2	131.2
14	25	75	1.5	95.53
15	25	80	2	141.19
16	25	80	2	131.58
17	25	85	2.5	209.3

**Table 3:** RSM analyses of variance.

Source	Sum of Squares	df	Mean Square	*F*-Value	*p*-Value	
Model	14,345.96	9	1594	7.16	0.0083	significant
A-GMA Concentration	1714.93	1	1714.93	7.70	0.0275	
B-Temperature	397.76	1	397.76	1.79	0.2231	
C-Time	11,590.03	1	11,590.03	52.06	0.0002	
AB	1.54	1	1.54	0.0069	0.9361	
AC	61.70	1	61.70	0.2772	0.6148	
BC	158.38	1	158.38	0.7115	0.4268	
A^2^	278.78	1	278.78	1.25	0.3000	
B^2^	29.61	1	29.61	0.1330	0.7261	
C^2^	128.55	1	128.55	0.5775	0.4721	
Residual	1558.28	7	222.61			
Lack of Fit	1167.75	3	389.25	3.99	0.1074	not significant
Pure Error	390.53	4	97.63			
Cor Total	15,904.24	16				

C.V.% = 10.5%; adjusted R^2^ = 0.7760.

**Table 4:** Kinetic parameters of adsorption by I-(PP-g-GMA-NMDG) fibers.

*q*_e,experiment_ (mg/g)	Pseudo-First-Order Model			Pseudo-Second-Order Model		
	*q*_e_ (mg/g)	k_1_ (min^−1^)	*R* ^2^	*q*_e_ (mg/g)	k_2_ (g/mg·min)	*R* ^2^
18.26	16.77	0.0392	0.9798	21.06	0.0029	0.9968

**Table 5:** Fitting parameters of the adsorption isotherm models for I-(PP-g-GMA-NMDG) fibers.

T (°C)	Langmuir			Freundlich			Temkin		
	*q*_m_	K_L_	*R* ^2^	*n*	K_F_	*R* ^2^	*B*_T_	K_T_	*R* ^2^
25	39.05	0.0095	0.9517	2.1986	2.1129	0.9909	10.0508	0.0627	0.9717
30	24.06	0.0224	0.9170	3.7015	4.394	0.9945	4.8014	0.2580	0.9762
35	18.83	0.0335	0.9408	5.1840	5.5864	0.9941	2.8902	1.1672	0.9936

**Table 6:** Adsorption properties of boron adsorbent materials.

Adsorbents	Substrate Material	*q*_e_ (mg/g)	References
Commercial resin D564		15.78	[38]
PGMA–PS-NMDG	PGMA-PS	21.66	[39]
CTS-NMDG	CCTS	20.36	[7]
Poly(Si-NMDG)@MIL-101(Cr)	MIL-101	24.80	[40]
Si-NMDG	SiO_2_	16.68	[41]
PAF-1-NMDG	PAF-1	17.51	[42]
CCTS-IBPG	CCTS	29.19	[43]
Mg-Al-CLDH		22.1	[44]
I-(PP-g-GMA-NMDG)	PP	35.85	This work

**Table 7:** Thermodynamic parameters.

T (°C)	∆G^0^ (kJ/mol)	∆H^0^ (kJ/mol)	∆S^0^ (kJ/mol·K)
25	3.615	−8.796	−0.0416
30	3.766		−0.0414
35	4.032		−0.0416

## Data Availability

The data are contained within this article.
